# Dietary encapsulated essential oils and organic acids mixture improves gut health in broiler chickens challenged with necrotic enteritis

**DOI:** 10.1186/s40104-019-0421-y

**Published:** 2020-02-21

**Authors:** Van Hieu Pham, Liugang Kan, Jinyu Huang, Yanqiang Geng, Wenrui Zhen, Yuming Guo, Waseem Abbas, Zhong Wang

**Affiliations:** 1 State Key Laboratory of Animal Nutrition, College of Animal Science and Technology, China Agricultural University, Beijing, 100193 China; 2 Faculty of Animal Science and Veterinary Medicine, Thai Nguyen University Agriculture and Forestry, Thai Nguyen, Vietnam; 3 Menon Animal Nutrition Technology Co. Ltd., Shanghai, China

**Keywords:** Broiler chickens, *Clostridium perfringens*, Essential oils and organic acids, Gut health

## Abstract

**Background:**

The poultry industry is in need of effective antibiotic alternatives to control outbreaks of necrotic enteritis (NE) due to *Clostridium perfringens*. In the present study, we investigated the effects of dietary supplementation with a blend of encapsulated essential oils and organic acids (BLJ) on growth performance and gut health using a coinfection model of NE in broiler chickens.

**Methods:**

Two hundred and eighty-eight one-day-old male Arbor Acres broiler chicks were randomly assigned using a 2 × 2 factorial design into two groups fed either 0 or 500 mg/kg dietary BLJ and co-challenged (or not challenged for the control) with *Eimeria* spp./*C. perfringens*.

**Results:**

Infected birds fed the BLJ-supplemented diet exhibited an improved feed conversion ratio throughout the trial (*P* < 0.01), a higher villus height and villus height/crypt depth ratio, and reduced intestinal *C. perfringens* counts, liver *C. perfringens* carriage, gut lesion scores and serum fluorescein isothiocyanate dextran (FITC-D) concentrations at 7 d post-infection compared with those of birds without BLJ supplementation (*P* < 0.05). NE-infected birds fed BLJ exhibited significantly upregulated claudin-1 and *IGF-2* mRNA levels (*P* < 0.05), increased *A20* mRNA expression and significantly downregulated *TRAF-6*, *TNFSF15* and *TOLLIP* mRNA levels in the jejunum at 7 d post-infection compared with those in birds without BLJ supplementation (*P* < 0.05). Compared with the uninfected and untreated birds, the uninfected birds fed BLJ displayed increased relative abundances of *Lactobacillus* and *Coprococcus* but reduced Rikenellaceae levels. Compared with the unsupplemented NE-challenged birds, infected birds fed BLJ showed an increased relative abundance of Unclassified_Lachnospiraceae and a significantly decreased relative abundance of Erysipelotrichaceae.

**Conclusion:**

BLJ supplementation improved growth performance and gut health in NE-infected broiler chickens by strengthening the intestinal barrier function, positively modulating the gut microbiota community and differentially regulating intestinal immune responses. Our results also suggested that adding BLJ effectively controlled NE infections after experimental *Eimeria* and *Clostridium perfringens* coinfection.

## Background

Necrotic enteritis (NE) due to *Clostridium perfringens* is an economically important disease in the poultry industry that is characterized by increased mortality, poor bird welfare and huge economic losses as reviewed by Timbermont et al. [1]. In recent decades, in-feed antibiotics were used relatively freely as growth promoters, which helped control NE incidence. However, the ban on using antibiotic growth promoters in poultry feed owing to growing concern about antibiotic-resistant bacteria and the transfer of antibiotic residues in meat and eggs has led to the frequent occurrence of enteric disorders, such as NE [2]. This ban and its consequences have shifted the research focus to exploring effective alternatives to antibiotic growth promoters that can help cost-effectively ameliorate enteric disorders.

Essential oils (EOs) are extracted from plant flowers, leaves, stems, roots, seeds or fruits by steam distillation, extrusion or solvent extraction [3]. The major component of many EOs are phenolic compounds (terpenoids and phenylpropanoids) such as thymol, carvacrol and eugenol [4]. *In vitro *studies have shown that EOs have antibacterial, antiviral, antifungal, antimycotic, antiparasitic, insecticidal, antioxidant, anti-inflammatory, antitoxigenic, antiquorum-sensing and immune-regulating properties as reviewed in previous reports [5–8]. Thymol and eugenol alter the membrane permeability of microorganisms, causing leakage of intracellular materials. This perturbation in the lipid fraction of the plasma membrane is suggested to generate antimicrobial action [7, 9]. In addition, thymol, eugenol and carvacrol are structurally similar and are reported to exert synergistic or additive antimicrobial effects when supplemented together, even at lower concentrations [4]. The *in vitro* minimum inhibitory concentration assay showed strong antibacterial activity of the EO product, thymol and carvacrol against pathogenic *Escherichia coli, C. perfringens* and *Salmonella* strains and weak activity towards beneficial *Lactobacillus* strains [9]. Therefore, EOs are receiving increasing attention as potential antibiotic growth promotor alternatives in animal production.

Many experiments have indicated that EO supplementation or blends in pig and chicken diets, especially during the grower phase, improved feed palatability and growth performance [10–20], stimulated digestive secretions for improved nutrient digestibility [20, 21] and regulated the gut microbiota compositions [21, 22] and lipid metabolism [23]. Additionally, some *in vivo* trials showed that when animals or poultry were challenged with pathogens, including *Salmonella* [15], pathogenic *E. coli* [15] and *C. perfringens*, or parasites, such as *Eimeria* spp*.* [10], EOs also exerted antimicrobial [11], antioxidant, anti-inflammatory [14] and antiparasitic activities, maintained intestinal integrity and strengthened mucosal barrier functions [24].

Organic acids (OAs), such as formic, acetic, propionic, sorbic, hexanoic, benzoic, caprylic and capric acids, are also widely used in livestock as antibiotic alternatives for their ability to improve growth performance, increase endogenous digestive enzyme secretion and activity, improve protein, amino acid and mineral element digestibility [25], benefit intestinal development, improve gut health, maintain the intestinal microecological balance, and exert antimicrobial activity against poultry pathogens such as *Escherichia coli* [22], *Salmonella* spp. [22], *Campylobacter jejuni* [26] and *C. perfringens* [27]. For example, in broiler chickens, adding coated sodium n-butyrate increased body weight gain and alleviated NE-associated gut injury by upregulating jejunal tight junction protein mRNA levels [28]. Challenged birds given an OAs blend containing formic, acetic, propionic, sorbic, caprylic and capric acids showed improved feed efficiency during the grower stage [29]. Medium-chain fatty acids, such as caproic acids, caprylic acids and capric acid, decrease the numbers of *Salmonella* in chickens [30, 31] and offer advantages for improving energy supply and performance in piglets, possibly also stabilizing the intestinal microbiota in the post-weaning period [32]. Benzoic acid can enhance the growth performance of weanling pigs by its effect on intestinal tract development [30], nutrient use [31], antioxidative properties and intestinal microbiota [30].

In recent years, the combined use of hydrophobic EOs with lipophilic OAs in broiler diets has been considered the most promising method of substituting antibiotics and has received much attention for the potential synergistic and additive benefits on growth performance and health in pigs and poultry compared with individual EOs or OAs [33]. A blend of OAs and EOs (EOAs) effectively controlled Salmonella in broiler chickens [34]. Adding the EOA mixture (sorbic acid, fumaric acid and thymol) during the grower phase increased efficiency, possibly by improving intestinal morphology and increasing digestive enzyme activities in broiler chickens [35]. Several studies have assessed the interaction effects between EOs and OAs, but these studies have yielded inconsistent results [36]. Sun et al. [18] reported that supplementation with dietary EOs (thymol and carvacrol) and an enzyme complex containing xylanase, glucanase and mannanase benefitted growth performance and gut health in broilers challenged with *C. perfringens*. However, information on the effects of substituting antibiotics with an EOA blend on the gut health of NE-infected broiler chickens is scarce.

BLJ, an EOA blend, is a compound product with 4% thyme, 4% carvacrol, 0.5% hexanoic acid, 3.5% benzoic acid and 0.5% butyric acid encapsulated in Ca-alginate and whey protein microcapsules. *In vitro* studies have demonstrated that encapsulated BLJ retains its antimicrobial activity (unpublished data). In addition, microcapsules with an optimized encapsulation formula showed the desired release of the above-mentioned EOs and OAs in a simulated intestinal model and enhanced delivery to the chicken and pig intestines (unpublished data). The present study assessed whether dietary supplementation with BLJ could effectively control NE infections in broiler chickens. The underlying mechanism of action was further investigated by determining the gut microbiota composition, intestinal barrier-related gene expression (claudin-1, *ZO-1* and occludin) and intestinal Toll-like receptor (*TLR*) immune-related gene expression in broiler chickens challenged with NE.

## Materials and methods

### Experimental design, birds and diets

A 2 × 2 completely randomized factorial design was used to investigate the effects of two levels of BLJ supplementation (0 and 500 mg/kg of diet) and two levels of NE challenge (NE-challenged or unchallenged). Two hundred and eighty-eight 1-day-old male broiler chicks were purchased from a commercial hatchery (Beijing Arbor Acres Poultry Breeding Company, Beijing, China). Chicks were randomly divided into the four experimental groups, and each group had six replicate pens with 12 birds per pen. The treatment groups were as follows: (i) negative control group (no BLJ supplementation, no NE infection, group A); (ii) BLJ-treated group (BLJ supplementation at 500 mg/kg from d 1–42, no NE, group B); (iii) NE-infected control group (no BLJ supplementation, challenged with NE, group D); and (iv) BLJ-treated and NE-infected group (BLJ supplementation at 500 mg/kg from d 1–42, challenged with NE, group G). The BLJ was provided by Menon Animal Nutrition Technology Co. Ltd., Shanghai, China. To avoid cross-contamination, the uninfected and NE-infected birds were reared in separate areas. In accordance with the AA Broiler Management Guide, all birds received continuous light for the first 24 h and were then maintained under a 23-h light/1-h dark cycle for the remainder of the study. The temperature in the pen was maintained at 33–34 °C for the first three days post-hatch, then gradually decreased by 2 °C per week to a final temperature of 22–24 °C. An antibiotic-free, coccidiostat-free, pelleted basal diet was prepared according to the National Research Council (NRC, 1994) requirements for the starter (d 1–21) and grower (d 22–42) periods. Table 1 presents the basal feed compositions and associated nutrient levels. Birds were provided feed and water ad libitum throughout the trial.

**Table 1:** Composition and nutrient levels of the experimental basal diet, on an as-fed basis unless stated otherwise, %

Items	1 to 21 d	22 to 42 d
Composition, %		
Corn (CP 7.8%)	39.70	57.0
Wheat powder	0	5
Wheat	19.0	0
Soybean meal (CP 46.0%)	33.0	30.0
Soybean oil	4.00	4.40
Limestone-calcium carbonate	1.50	1.50
Calcium hydrogen phosphate	1.50	1.36
Phytase	0.02	0.03
*DL*-Methionine, 98%	0.27	0.19
*L*-Lysine HCl, 78%	0.20	0.11
Sodium chloride	0.30	0.30
Vitamin premix^1^	0.03	0.03
Mineral premix^2^	0.20	0.20
Choline chloride, 50%	0.25	0.15
Ethoxyquin, 33%	0.05	0.03
Total	100	100
Calculated nutrient levels^3^		
Metabolizable energy, kcal/kg	3015.55	3101.20
Crude protein, %	21.37	19.27
Calcium, %	0.99	0.93
Available phosphorus %	0.45	0.43
Lysine, %	1.20	1.05
Methionine, %	0.57	0.46
Methionine + Cysteine, %	0.90	0.78

^1^Vitamin premix provided per kg of complete diet: vitamin A (retinyl acetate), 12,500 IU; vitamin D_3_ (cholecalciferol), 2,500 IU; vitamin E (*DL*-a-tocopherol acetate), 30 IU; vitamin K_3_ (menadione sodium bisulfate), 2.65 mg; vitamin B_12_ (cyanocobalamin), 0.025 mg; biotin, 0.30 mg; folic acid, 1.25 mg; nicotinic acid, 50 mg; *D*-pantothenic acid, 12 mg; pyridoxine hydrochloride, 6.0 mg; riboflavin, 6.5 mg; thiamine mononitrate, 3.0 mg

^2^Mineral premix provided per kg of complete diet: iron, 80 mg; copper, 8 mg; manganese, 100 mg; zinc, 80 mg; iodine, 0.35 mg; selenium, 0.15 mg

^3^Calculated value based on analysis of the experimental diets

### Necrotic enteritis disease model

NE was induced in broilers as previously described with some modifications [37]. Briefly, birds in the challenged groups were orally gavaged via the crop with *Eimeria maxima* (1.0 × 10^4^ oocysts/bird) and *Eimeria necatrix* (5.0 × 10^3^ oocysts/bird) oocysts (received from Prof. Suoxun, College of Veterinary Medicine, China Agricultural University) on day 14 post-hatch followed by oral gavage with 1 mL of *C. perfringens* type A CVCC52 (China Veterinary Culture Collection Center, China Institute of Veterinary Drug Control, Beijing, China) at 2.2 × 10^8^ colony-forming units (CFU)/mL per day from d 18–20. Uninfected control birds received 1 mL of sterile phosphate-buffered saline by oral gavage at the same time-points. Feed was withdrawn 8 h prior to each inoculation.

### Growth performance

Body weight (BW) and feed intake from each replicate cage were measured on d 1, 21 and 42. Average body weight gain (BWG), average feed intake (AFI) and the feed conversion ratios (FCRs) were calculated. Mortality rates were recorded daily.

### Intestinal lesion scores and sample collection

At 7 d post-infection (DPI; at 28 d of age) with *C. perfringens*, one bird per replicate was randomly selected, weighed and euthanized via cervical dislocation. Three independent observers blinded to the study groups scored the NE gut lesions on a scale of 0 (no lesions) to 4 (severe lesions) as previously described [28]. Concurrently, ∼1-cm-long jejunal samples taken from between Meckel’s diverticulum and the proximal end of the jejunum were snap-frozen in liquid nitrogen and stored at − 80 °C for mRNA analysis. Additional ∼2-cm-long jejunal samples, taken midway between the endpoint of the duodenal loop and Meckel’s diverticulum, were collected, flushed with 10% neutral buffered formalin and fixed overnight in 10% neutral buffered formalin for histological examination. The cecal contents and liver tissues were aseptically collected and immediately frozen at − 40 °C for bacterial population analysis or bacterial translocation analysis, respectively.

### Histomorphological structure and goblet cell analysis of the jejunum

The gut histomorphology (villus height and crypt depth) and goblet cells were analyzed as previously described [38]. Briefly, the fixed tissue samples were dehydrated in a tissue processor (Leica Microsystems K. K., Tokyo, Japan) and embedded in paraffin wax. Paraffin sections (5 μm) were sliced using a microtome (Leica Microsystems K. K., Tokyo, Japan) and mounted on glass slides. The paraffin was removed by xylene (twice for 5 min each), followed by rehydration in 95% alcohol (5 min) and 50% alcohol (5 min). Sections were stained with hematoxylin and eosin (H&E) for villous morphology measurement (Leica Microsystems Ltd., Wetzlar, Germany). Goblet cells were visualized by periodic acid–Schiff staining. The area of the goblet cells in different intestinal sections was counted based on the length and width of the goblet cell “cup” in cross-sections of the villi under an Olympus light microscope (Olympus Optical Co., Beijing, China). The density of goblet cells was calculated as the number of goblet cells per unit surface area (mm^2^).

### Intestinal permeability analysis by measuring bacterial translocation and serum fluorescein isothiocyanate dextran (FITC-D) concentrations

*C. perfringens* cells in the liver were counted using the plate-pouring method as previously described [37]. Bacterial translocation was expressed in CFUs (log_10_ CFU/g of tissue). At 7 DPI, all chickens were orally gavaged with FITC-D (3000–5000 Da molecular weight, Sigma Aldrich, St. Louis, MO, USA) at 8.32 mg/mL/bird. Blood samples were collected at 1 or 2.5 h after administering FITC-D, then centrifuged at 3000 × *g* for 10 min to separate the serum for FITC-D analysis as previously described [39]. In brief, standard curves (0, 0.0001, 0.001, 0.01, 0.1, 1.0 and 10 μg/mL) were prepared using FITC-D. FITC-D levels in diluted sera (1:5) were measured at excitation and emission wavelengths of 485 nm and 528 nm, respectively (Synergy HT, multi-mode microplate reader, BioTek Instruments, Inc., VT, USA). The FITC-D concentration per mL of serum was calculated based on a standard curve.

### Real-time polymerase chain reaction (PCR)

Total RNA was isolated from the snap-frozen jejunal tissue samples (50 mg) with an RNeasy mini kit following the animal tissue protocol (Qiagen, Germantown, MD, USA). The purity and concentration of the total RNA were measured with a spectrophotometer (NanoDrop-2000, Thermo Fisher Scientific, Waltham, MA, USA) using a 260:280-nm absorbance ratio. The absorption ratios (OD_260_/OD_280_) of all of the samples ranged from 1.8–2.0. First-strand cDNA was synthesized from 2 μg of total RNA using a Primer Script™ RT reagent kit with gDNA Eraser (Perfect Real Time; Takara Biotechnology Co. Ltd., Tokyo, Japan) as per the manufacturer’s instructions and stored at − 80 °C until further processing. Quantitative real-time PCR (qRT-PCR) oligonucleotide primers for the TLR signaling pathway-related genes, tight junction proteins, growth factors, mucin-2 and β-actin (Tables 2 and 3) were designed with Primer Express 3.0 (Applied Biosystems, Foster City, CA, USA) and synthesized by Sangon Biotech Co., Ltd. (Shanghai, China). Primers were designed to span introns to avoid genomic DNA amplification. Quantitative real-time PCR was performed using the Applied Biosystems 7500 Fast Real-Time PCR System and a SYBR Premix Ex Taq™ kit (Takara Biotechnology Co. Ltd., Beijing, China). Reactions were performed in a 20-μL volume containing 10.0 μL of SYBR Premix Ex Taq (2×) mix, 1.0 μL of cDNA, 0.5 μL of each primer (10 mM) and 8.0 μL of sterile nuclease-free water. For the PCR, samples were subjected to an initial denaturation phase at 95 °C for 5 min, followed by 40 cycles of denaturation at 95 °C for 30 s and annealing and extension at 60 °C for 30 s. Melt curve analysis was performed to confirm the PCR amplification specificity. All tissue samples for cDNA synthesis were analyzed in triplicate. All PCR amplifications were performed in triplicate. Gene expressions were analyzed using β-actin as an internal control. Average gene expression relative to the β-actin internal control for each sample was calculated using the 2^–ΔΔCt^ method [40].

**Table 2:** Nucleotide sequences of primers (TLR-mediated signaling pathway-related cytokines, chemokines and negative regulators) for quantitative real-time PCR^1^ assay

Name		Primer sequence (5' → 3')	GenBank accession
Receptors	*TLR-2*	F: GGGGCTCACAGGCAAAATC	NM_001161650.1
		R: AGCAGGGTTCTCAGGTTCACA	
	*TLR-4*	F:CCACTATTCGGTTGGTGGAC	NM_001030693.1
		R:ACAGCTTCTCAGCAGGCAAT	
Adaptor proteins	*MyD88*	F:GGATGGTGGTCGTCATTTCA	NM_001030962.1
		R:GAGATTTTGCCAGTCTTGTCCA	
	*TRAF-6*	F: CACAGAGGAGACGCAGGGATA	XM_001235884.1
		R: AACAGATCGGGCACTCGTATTT	
	*NF-kB*	F:TGGAGAAGGCTATGCAGCTT	NM_205134.1
		R:CATCCTGGACAGCAGTGAGA	
Pro-inflammatory cytokines	*TNFSF15*	F- CCAAGAGCACACCTGACAGT	NM_001024578.1
		R- CACAGGTATCACCAGTGCGT	
	*IL-1β*	F-CAGCAGCCTCAGCGAAGAG	NM_204524.1
		R-CTGTGGTGTGCTCAGAATCCA	
	*IL-8*	F-GGCTTGCTAGGGGAAATGA	AJ009800
		R-AGCTGACTCTGACTAGGAAACTGT	
	*IFN-γ*	F-AAAGCCGCACATCAAACACA	NM_205149.1
		R-GCCATCAGGAAGGTTGTTTTTC	
Ant-inflammatory cytokines	*IL-10*	F:CGCTGTCACCGCTTCTTCA	NM_001004414.2
		R:CGTCTCCTTGATCTGCTTGATG	
Negative regulators	*Tollip*	F:CATGGTACCTGTGGCAATACC	NM_001006471
		R:GCACTGAGCGGATTACTTCC	
	*PI3K*	F:AACATCTGGCAAAACCAAGG	NM_001004410
		R:CTGCAATGCTCCCTTTAAGC	
	*A20*	F:GAGAACGCAGAGCCTACACC	NM_001277522.1
		R:CCAACCTTCTTCCTGCACAT	
	*SOCS-1*	F:GCTCTCAGGCTCGAGGTTAC	NM_001137648.1
		R:GCTTGCTCGAGTGATGCTACT	
	*SOCS-6*	F: CAGATATCTTTGTGGACCAGGCAGTGAA	NM_001127312
		R: GGTAGCAAAGGTGAAAGTGGAGGGACATC	

^1^Primers were designed and synthesized by Sango Biotech (Shanghai) Co., Ltd. F: forward; R: reverse

*TLR* Toll-like receptor; *MyD88* myeloid differential protein-88; *TRAF-6* TNF receptor-associated factor 6; *NF-κB* nuclear factor kappa-light-chain-enhancer of activated B cells; *TNFSF15* tumor necrosis factor superfamily member 15; *IL* interleukin; *IFN-γ* interferon γ; *Tollip* Toll-interacting protein; *PI3K* phosphatidylinositol 3-kinase; *A20* protein A20; *SOCS* suppressor of cytokine signaling

**Table 3:** Nucleotide sequences of primers (tight junction proteins and growth factors) for quantitative real-time PCR^1^ assay

Name	Primer sequence (5´→3´)	GenBank accession
Tight junctions		
Claudin-1	F: AAGTGCATGGAGGATGACCA	NM_001013611.2
	R: GCCACTCTGTTGCCATACCA	
Occludin	F:TCATCGCCTCCATCGTCTAC	NM_205128.1
	R:TCTTACTGCGCGTCTTCTGG	
*ZO-1*	F: TATGAAGATCGTGCGCCTCC	XM_015278981.1
	R: GAGGTCTGCCATCGTAGCTC	
Mucin-2	F: AGCGAGATGTTGGCGATGAT	NM_001318434.1
	R: AAGTTGCCACACAGACCACA	
Growth factors		
*TGF-β3*	F:TGCGGCCAGATGAGCAT	NM_205454.1
	R:TGCACATTCCTGCCACTGA	
*IGF-2*	F: TGGCTCTGCTGGAAACCTAC	NM_001030342.2
	R: ACTTGGCATGAGATGGCTTC	
*EGFR*	F: ACCAGCCTGCAGAGAATGTA	NM_205497
	R: CACCATGTTAAGCGCAATGA	
*GLP-2*	F:AAGCTTCCCAGTCTGAACCA	NM_205260.4
	R:ATCCTGAGCTCGTCTGCTGT	
House-keeping genes		
β-actin	F: GAGAAATTGTGCGTGACATCA	NM 205518
	R: CCTGAACCTCTCATTGCCA	

^1^Primers were designed and synthesized by Sango Biotech (Shanghai) Co., Ltd. F: forward; R: reverse

*ZO-1* zonula occludens-1; *EGFR* epidermal growth factor receptor; *GLP-2* glucagon-like peptide-2; *IGF-2* insulin-like growth factor-2; *TGF- β3* transforming growth factor beta 3

### Microbiota DNA extraction, 16S rRNA amplification, sequencing and sequence data processing

Microbial genomic DNA was extracted from cecal content samples using the QIAamp Fast DNA stool mini kit (Qiagen, Mannheim, Germany) following the manufacturer’s instructions. The quantity and quality of the extracted DNA were measured using a NanoDrop ND-1000 spectrophotometer (Thermo Fisher Scientific) and agarose gel electrophoresis, respectively, then the genomic DNA was used as a template for PCR amplification. The bacterial 16S RNA V3–V4 gene region was amplified using the KAPA HiFi Hotstart Ready Mix PCR kit (Kapa Biosystems, Wilmington, Massachusetts, USA) and primers F341 and R806 (F341: 5′-ACTCCTACGGGAGGCAGCA-3′, R806: 5′-GGACTACHVGGGTWTCTAAT-3′). The PCR conditions were as follows: initial pre-denaturation at 94 °C for 5 min, 30 cycles of denaturation at 95 °C for 30 s, annealing at 50 °C for 30 s, elongation at 72 °C for 30 s and a final elongation at 72 °C for 5 min. The amplicons were examined via 2% agarose gel electrophoresis, and the target fragment was excised and subsequently purified using a QIA quick Gel Extraction Kit (Qiagen, USA). Finally, 16S rRNA gene sequencing was performed using the Illumina MiSeq PE250 platform (Illumina, Santa Clara, CA, USA) with the MiSeq Reagent Kit at Shanghai Personal Biotechnology Co., Ltd. (Shanghai, China).

The raw sequence data obtained from the Illumina MiSeq platform were quality-filtered and demultiplexed using Quantitative Insights into Microbial Ecology (QIIME), version 1.8.0-dev (http://qiime.org/index.html). Sequences with an average Phred score lower than 20, containing ambiguous bases, homopolymer runs exceeding six, mismatched primers or sequence lengths shorter than 150 bp were removed. Only sequences with an overlap longer than 10 bp and with no mismatches were assembled according to their overlap sequence using FLASH [41]. Reads that could not be assembled were discarded. Barcode and sequencing primers were trimmed from the assembled sequence. Trimmed sequences were uploaded to QIIME for further analysis. The trimmed and assembled sequences from each sample were aligned to the Greengenes 16S rRNA training set 10 using the best hit classification option to classify the taxonomic abundance in QIIME [42]. Bacterial operational taxonomic units (OTUs) were generated using the UCLUST function in QIIME (http://qiime.org/scripts/pick_otus) with a 97% similarity threshold. The alpha diversity measures, including the observed OTUs/read and the abundance-based coverage (ACE), Chao 1, Good’s coverage, Shannon and Simpson indices were calculated using MOTHUR. Beta diversity analysis was performed to investigate the structural variation of microbial communities across samples using UniFrac distance metrics [43]. Differences in the UniFrac distances for pairwise comparisons among groups were determined using the Student’s t-test and Monte Carlo permutation test with 1000 permutations and were visualized through box-and-whisker plots. Principal component analysis was conducted based on the genus-level compositional profiles [44]. A Venn diagram was generated to visualize the shared and unique OTUs among samples or groups using the R package, “Venn Diagram”, based on OTU occurrence across samples/groups regardless of their relative abundances [45]. Partial least squares discriminant analysis (PLS-DA) was also introduced as a supervised model to reveal the microbiota variation among groups using the “PLS-DA” function in the R package, “mix Omics” [46]. Significant differences between the microbiota compositions in the control and BLJ-treated chickens were determined via a nonparametric Mann–Whitney U test ranked using the percentage of representation of individual genera.

### Statistical analysis

Data regarding growth performance, gut lesion scores, intestinal bacterial concentrations, liver *C. perfringens*, jejunum morphology, goblet cell numbers, intestinal permeability, relative mRNA expression levels and the Shannon and ACE alpha diversity indices among the four groups were analyzed using one-way ANOVA in SPSS 20.0 (SPSS Inc., Chicago, IL, USA) in a 2 × 2 factorial design. Mean separations were conducted using Duncan’s multiple comparison when interactive effects differed significantly. Phylum and genus abundances were compared using the Kruskal–Wallis test with Benjamini–Hochberg *P*-value correction. *P* ≤ 0.05 was considered significant, and 0.05 ≤ *P* ≤ 0.10 was considered a trend.

## Results

### Growth performance

Table 4 presents the growth performance results for the broiler chickens. Compared with the unchallenged groups, the untreated NE-challenged group had significantly decreased BWG on d 1 to 21 and d 1 to 42, and AFI from d 1 to 21, and notably increased FCR at the different stages (*P* < 0.01). Dietary supplementation with BLJ resulted in a notable improvement in FCR (*P* < 0.01), whereas a significantly reduced AFI (*P* < 0.05) at the later and whole trial stage compared with the unsupplemented group.

**Table 4:** Effect of BLJ on growth performance of broiler chickens challenged with NE

Items	Experimental design				SEM^5^	Main effect				*P*-value^6^		
	A^1^	D ^2^	B^3^	G^4^		0	500	Non-challenge	Challenged	Treatment	Challenged	T×C
d 1 to 21												
BWG, g/bird	870	655	857	647	21.40	763	752	864^a^	651^b^	0.449	< 0.01	0.843
AFI, g/bird	1249	1047	1246	1057	20.14	1148	1151	1248^a^	1052^b^	0.842	< 0.01	0.676
FCR, g/g	1.44	1.60	1.45	1.64	0.02	1.52	1.55	1.44^b^	1.62^a^	0.085	< 0.01	0.533
d 22–42												
BWG, g/bird	1904	1891	1883	1919	29.59	1898	1901	1894	1905	0.955	0.844	0.681
AFI, g/bird	3095^a^	3114^a^	2684^b^	3083^a^	39.40	3104	2883	2890	3098	0.010	0.015	0.025
FCR, g/g	1.63^a^	1.65^a^	1.43^b^	1.60^a^	0.01	1.64	1.51	1.53	1.62	< 0.01	< 0.01	< 0.01
d 1 to 42												
BWG, g/bird	2774	2546	2740	2566	34.08	2660	2653	2757^a^	2556^b^	0.918	0.008	0.696
AFI, g/bird	4344	4161	3931	4140	55.89	4252^a^	4035^b^	4137	4151	0.033	0.890	0.050
FCR, g/g	1.54^b^	1.63^a^	1.44^c^	1.62^a^	0.01	1.59	1.53	1.50	1.63	< 0.01	< 0.01	< 0.01

^a, b^ Means within the same row without a common superscript differ significantly (*P* < 0.05)

^1^*A* neither BLJ treatment nor NE infection

^2^*D* NE infection but without BLJ treatment

^3^*B* BLJ treatment at 500 mg/kg of feed but without NE infection

^4^*G* both BLJ treatment and NE infection

^5^*SEM* standard error of the mean

^6^*P*-values represent the main effect of the diet, the main effect of the NE challenge, and the interaction between the dietary treatments and NE challenge; BWG: body weight gain, g/bird; AFI: average feed intake, g/bird; FCR: feed conversion ratio, g of feed intake/g of BW gain, g/g; T×C: treatment and challenged

A notable interaction was observed for FCR and AFI during d 22 to 42 and over the whole period between BLJ administration and NE challenge. Non-infected birds fed BLJ displayed a significant reduction in AFI (*P* ≤ 0.05) and a remarkable improvement in FCR (*P* < 0.01) compared with the single NE-challenged control and other treatments.

### Intestinal lesion scores and morphological observations

NE infection significantly increased the jejunum crypt depth (*P* < 0.01) and the small intestinal lesion scores (*P* < 0.05), reduced the villus height (*P* = 0.076) and remarkably decreased the VH/CD ratio in the jejunums of the NE-challenged birds compared with those of the uninfected birds (Table 5). In addition, chickens who received BLJ diets had greater villus height (*P* < 0.05) and VH/CD ratios (*P* < 0.01) in the jejunum compared with those of the unsupplemented groups (Table 5). Infected birds fed diets with BLJ also displayed lower gut lesion scores (*P* < 0.05) in the small intestines at 7 DPI (d 28) than those of the untreated NE-infected birds, and lower crypt depths (*P* < 0.05) compared with those of birds that did not receive BLJ-supplemented diets. The interaction between BLJ supplementation and NE infection had a combined effect on the small intestinal lesion score, crypt depth and VH/CD ratio at d 28 (7 DPI). NE-infected birds fed diets supplemented with BLJ exhibited significantly decreased gut lesion scores and crypt depths (*P* < 0.05) and a notably increased (VH/CD) ratio (*P* < 0.05) in the small intestine at 7 DPI compared with those of the single NE-infected birds. However, jejunal goblet cells (on d 28) did not significantly differ among the groups.

**Table 5:** Effect of dietary BLJ supplementation on jejunal lesion scores, morphology and goblet cell numbers in broiler chickens challenged with NE at 28 d of age

Items	NE^1^	Jejunum lesion scores	Villous height, μm	Crypt depth, μm	VH/CD^2^	GC^3^ cells
BLJ, mg/kg						
0	–	0.14^c^	439.66	61.93^b^	7.07^b^	23.57
0	+	1.33^a^	398.69	112.52^a^	3.56^c^	17.23
500	–	0.29^bc^	507.07	59.00^b^	8.66^a^	23.03
500	+	0.58^b^	446.39	66.69^b^	6.75^b^	23.87
SEM^4^		0.07	15.00	4.91	0.41	1.06
Main effect						
0		0.74	419.18^b^	87.22	5.31	20.40
500		0.43	476.73^a^	62.85	7.70	23.45
Non-challenged		0.21	473.37	60.47	7.86	23.30
Challenged		0.96	422.54	89.60	5.15	20.55
*P*-value^5^						
BLJ		0.043	0.046	< 0.01	< 0.01	0.130
Challenged		< 0.01	0.076	< 0.01	< 0.01	0.170
Challenged×BLJ		< 0.01	0.720	< 0.01	0.011	0.078

^a, b, c^Means within the same column with different superscripts differ significantly (*P* < 0.05)

^1^*NE* Co-challenged with *Eimeria* spp. and *Clostridium perfringens*; −, without NE challenge; +, with NE challenge

^2^*VH/CD* villus height/crypt depth ratio

^3^*GC cells* goblet cell numbers per mm^2^

^4^*SEM* standard error of the mean

^5^*P*-values represent the main effect of the diet, the main effect of the NE challenge, and the interaction between the dietary treatments and NE challenge

### Liver *C. perfringens* invasion and serum FITC-D levels

A significant interaction effect occurred between liver *C. perfringens* invasion and cecal *C. perfringens* colonization between the NE-infected and BLJ-supplemented groups (Table 6). Challenged birds fed diets supplemented with BLJ showed significantly fewer *C. perfringens* (*P* < 0.05) in the liver and cecal contents throughout the infection period compared with those in the NE-infected birds. The number of *C. perfringens* in the liver and cecum of the NE-infected birds at 7 DPI notably increased (*P* < 0.01) compared with those of the uninfected birds. Conversely, the *C. perfringens* populations in the livers and ceca of the BLJ-treated birds at 7 DPI decreased significantly (*P* < 0.01) compared with those of the unsupplemented group. The interaction between BLJ supplementation and NE infection had a combined effect on the serum FITC-D concentration at 1 h post-FITC-D gavage (Table 6). Compared with NE-infected birds and the untreated groups, the infected and uninfected birds fed BLJ exhibited lower serum FITC-D concentrations at 1 h post-FITC-D gavage (*P* < 0.05) but no significant effect was observed on the serum FITC-D concentration at 2.5 h post-FITC-D gavage.

**Table 6:** Effects of dietary supplementation with BLJ on serum FITC-D concentration and cecal and liver *Clostridium perfringens* (CFU/g) numbers in broiler chickens challenged with NE

Items	NE^1^	FITC-D, ng/mL		Liver *Clostridium perfringens,* CFU/g	Cecal *Clostridium perfringens,* CFU/g
		1 h	2.5 h		
BLJ, mg/kg					
0	–	9.74^ab^	9.84	0.32^b^	0.00^c^
0	+	9.88^a^	10.00	1.93^a^	4.69^a^
500	–	9.68^bc^	9.88	0.00^b^	0.00^c^
500	+	9.52^c^	9.83	0.42^b^	2.23^b^
SEM^2^		0.04	0.05	0.18	0.40
Main effect					
0		9.81	9.92	1.13	2.34
500		9.60	9.86	0.21	1.11
Non-challenged		9.71	9.86	0.16	0.00
Challenged		9.70	9.92	1.18	3.46
*P*-value^3^					
BLJ		< 0.01	0.552	< 0.01	< 0.01
Challenged		0.838	0.570	< 0.01	< 0.01
Challenged×BLJ		0.033	0.292	0.021	< 0.01

^a, b, c^ Means within the same column with different superscripts differ significantly (*P* < 0.05)

^1^*NE* Co-challenged with *Eimeria* spp. and *Clostridium perfringens*; −, without NE challenge; +, with NE challenge

^2^*SEM*, standard error of the mean

^3^*P*-values represent the main effect of the diet, the main effect of the NE challenge, and the interaction between the dietary treatments and NE challenge

### Expression of the intestinal tight junction and mucin-2 genes

Table 7 shows the changes in tight junction, mucin-2 and growth factor mRNA expression in the jejunum. Based on the main-effect NE challenge, NE infection notably downregulated occludin, zonula occludens-1 (*ZO-1*), epithelial cell growth factor receptor (*EGFR*) and mucin-2 mRNA levels and remarkably upregulated *GLP-2* and *IGF-2* mRNA levels in the jejunum (*P* < 0.05) compared with those in the unchallenged groups (*P* < 0.05). Conversely, BLJ-treated birds showed lower *ZO-1* and higher *IGF-2* and *GLP-2* expression levels in the jejunum than those in the unsupplemented controls. In addition, a significant interaction effect on *claudin-1, IGF-2* and *mucin-2* mRNA expressions occurred between NE infection and BLJ addition. Challenged birds fed diets supplemented with BLJ showed significantly higher *claudin-1* and *IGF-2* mRNA levels (*P* < 0.05) in the jejunum at 7 DPI compared with those in the NE-infected birds. In addition, uninfected birds fed BLJ-supplemented diets showed the highest levels of mucin-2 gene expression in the jejunum compared with those of the other three treatments.

**Table 7:** Effects of dietary supplementation with BLJ on gene expressions of tight junction proteins, growth factors and mucin-2 in the jejunums of broiler chickens challenged with NE (at 7 days after NE infection)

Items	NE^1^	Claudin1	Occludin	*ZO-1*	*TGF-β3*	*IGF-2*	*EGFR*	*GLP-2*	Mucin-2
BLJ, mg/kg									
0	–	0.82^b^	1.02	1.07	1.03	1.04^b^	1.02	1.04	1.05^b^
0	+	0.56^b^	0.93	0.66	0.89	1.34^b^	0.86	1.05	0.71^c^
500	–	0.50^b^	1.10	0.64	1.18	1.21^b^	1.45	1.26	1.44^a^
500	+	1.02^a^	0.57	0.21	1.17	2.47^a^	0.60	1.84	0.55^c^
SEM^2^		0.08	0.08	0.08	0.08	0.14	0.11	0.10	0.09
Main effect									
0		0.69	0.98	0.87^a^	0.96	1.19	0.94	1.05^b^	0.88
500		0.76	0.83	0.43^b^	1.17	1.84	1.03	1.55^a^	1.00
Non-challenged		0.66	1.06^a^	0.86^a^	1.10	1.13	1.24^a^	1.15	1.23
Challenged		0.79	0.57^b^	0.44^b^	1.03	1.90	0.73^b^	1.45	0.63
*P*-value^3^									
BLJ		0.592	0.310	< 0.01	0.194	< 0.01	0.669	0.003	0.322
Challenged		0.335	0.034	< 0.01	0.655	< 0.01	0.017	0.056	< 0.01
Challenged×BLJ		0.010	0.109	0.929	0.667	< 0.01	0.094	0.064	0.027

^a, b, c^ Means within the same column with different superscripts differ significantly (*P < 0.05*)

^1^*NE* Co-challenged with *Eimeria* spp. and *Clostridium perfringens*; −, without NE challenge; +, with NE challenge

^2^*SEM*, standard error of the mean

^3^*P*-values represent the main effect of the diet, the main effect of the NE challenge, and the interaction between the dietary treatments and NE challenge

### mRNA levels of TLR signaling-related cytokines and growth factors in the jejunum

NE infection significantly downregulated the *TLR-4*, *TRAF-6*, *NF-κB*, *TNFSF15*, *TOLLIP*, *PI3K* and *SOCS-6* mRNA levels (*P* < 0.05) and remarkably upregulated *IFN-γ* mRNA levels in the jejunum (*P* < 0.05) compared with those of the unchallenged groups (Table 8). The infected birds fed BLJ exhibited lower *TLR-4* and *TRAF-6* mRNA levels (*P* < 0.05), increased *A20* mRNA levels (0.05 < *P* < 0.10) and decreased *IL-1β* gene expression levels (0.05 < *P* < 0.10) compared with the unsupplemented groups. A dramatic interaction effect (*P* < 0.05) on *TLR-2*, *TRAF-6*, *TNFSF15*, *TOLLIP* and *SOCS-6* mRNA levels (*P* < 0.05) occurred between NE infection and BLJ addition. NE-infected birds fed diets supplemented with BLJ also exhibited significantly decreased *TRAF-6*, *TNFSF15* and *TOLLIP* gene expression levels and a decreasing trend was observed in the *TLR-2* mRNA level in the jejunum at 7 DPI compared with that in the uninfected birds. In addition, the uninfected birds fed BLJ had the highest *SOCS-6* gene expression levels in the jejunum at 7 DPI compared with those of the other three groups.

**Table 8:** Effects of dietary supplementation with BLJ on gene expressions of proinflammatory cytokines, chemokines and TLR signaling pathway-related genes in the jejunums of broiler chickens challenged with NE (at 7 d after NE infection)

Items	NE^1^	*TLR-2*	*TLR-4*	*MyD88*	*TRAF-6*	*NF-kB*	*IFN-γ*	*TNFSF15*	*IL-1β*	*IL-8*	*IL-10*	*Tollip*	*PI3K*	*A20*	*SOCS-1*	*SOCS-6*
BLJ, mg/kg																
0	–	1.01^ab^	1.66	1.02	1.02^a^	1.06	1.01	1.00^bc^	1.05	1.38	1.07	1.02^ab^	1.04	1.10	1.06	1.02^ab^
0	+	1.26^ab^	0.88	1.01	0.96^ac^	0.90	3.89	1.08^b^	1.14	0.63	1.37	0.84^b^	0.73	0.93	1.23	0.92^b^
500	–	1.45^a^	0.59	1.11	1.13^a^	1.16	1.13	1.32^a^	1.02	0.75	0.81	1.16^a^	1.26	1.35	1.07	1.45^a^
500	+	0.91^b^	0.46	0.87	0.36^b^	0.39	3.13	0.79^c^	0.62	1.02	1.24	0.57^c^	0.73	1.70	1.19	0.67^b^
SEM^2^		0.08	0.13	0.04	0.08	0.10	0.34	0.05	0.08	0.14	0.11	0.06	0.07	0.14	0.11	0.09
Main effect																
0		1.14	1.27^a^	1.02	0.99	0.98	2.45	1.04	1.10	1.01	1.22	0.93	0.88	1.01	1.15	0.97
500		1.18	0.52^b^	0.99	0.74	0.78	2.13	1.06	0.82	0.89	1.02	0.86	1.00	1.53	1.13	1.06
Non-challenged		1.23	1.13^a^	1.07	1.07	1.11^a^	1.07^b^	1.16	1.04	1.07	0.94	1.09	1.15^a^	1.22	1.07	1.23
Challenged		1.09	0.67^b^	0.94	0.66	0.64^b^	3.51^a^	0.93	0.88	0.83	1.30	0.70	0.73^b^	1.32	1.21	0.80
*P*-value^3^																
BLJ		0.780	< 0.01	0.788	0.018	0.219	0.500	0.843	0.085	0.663	0.360	0.323	0.324	0.078	0.957	0.561
Challenged		0.339	0.017	0.118	< 0.01	< 0.01	< 0.01	< 0.01	0.316	0.381	0.107	< 0.01	< 0.01	0.737	0.525	0.013
Challenged×BLJ		0.013	0.081	0.162	< 0.01	0.068	0.358	< 0.01	0.125	0.073	0.775	< 0.01	0.319	0.361	0.914	0.044

^a, b, c^ Means within the same column with different superscripts differ significantly (*P* < 0.05)

^1^*NE* Co-challenged with *Eimeria* spp. and *Clostridium perfringens*; −, without NE challenge; +, with NE challenge

^2^*SEM* standard error of the mean

^3^*P*-values represent the main effect of the diet, the main effect of NE challenged, and the interaction between the dietary treatments and NE challenged

### Cecal microbiome

**Fig. 1:**
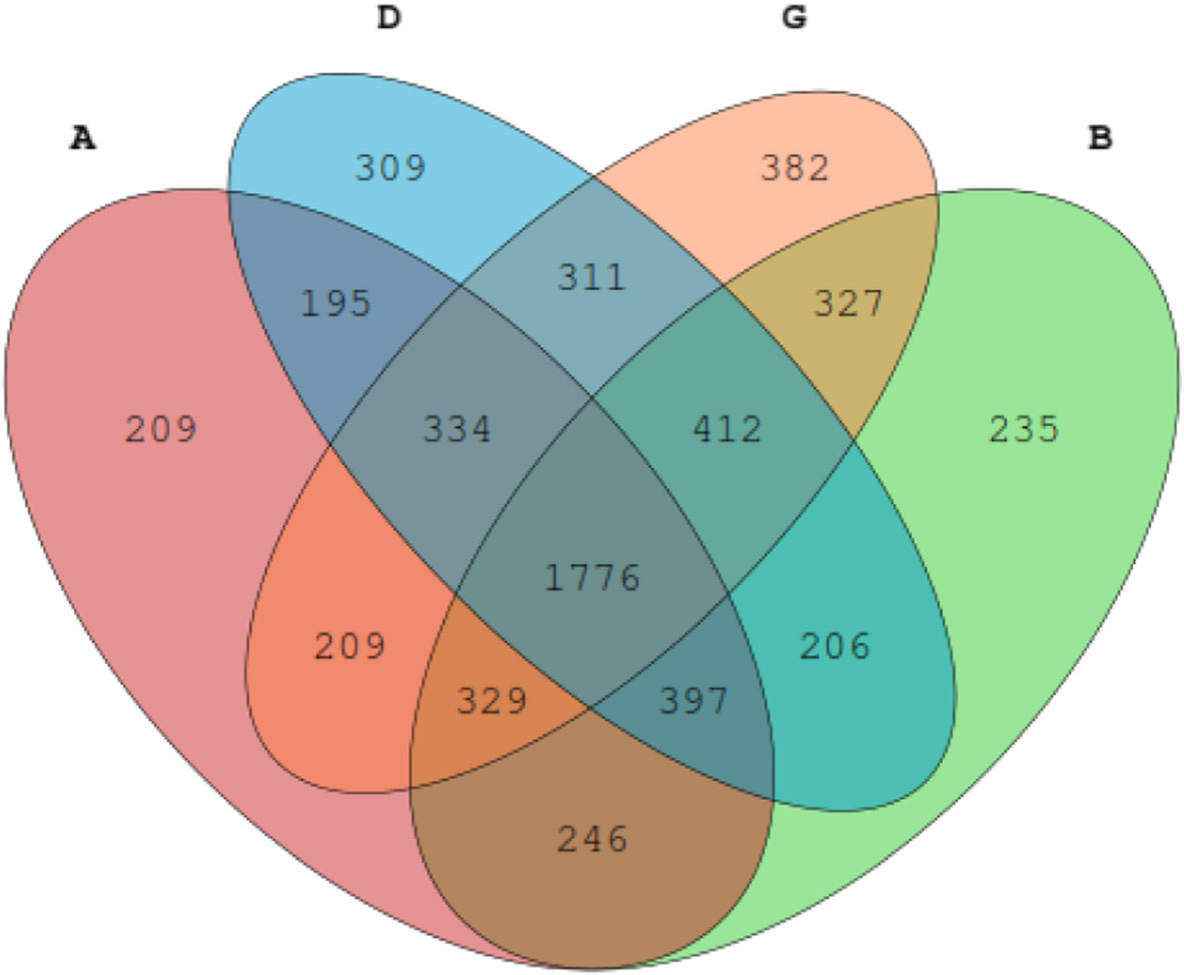
Venn diagram illustrated the number of common and unique core OTUs among the four groups. **a** = a basal diet + unchallenged; **b** = a basal diet with 500 mg/kg of BLJ + unchallenged; **d** = a basal diet + challenged; and **g** = a basal diet with 500 mg/kg of BLJ

**Fig. 2:**
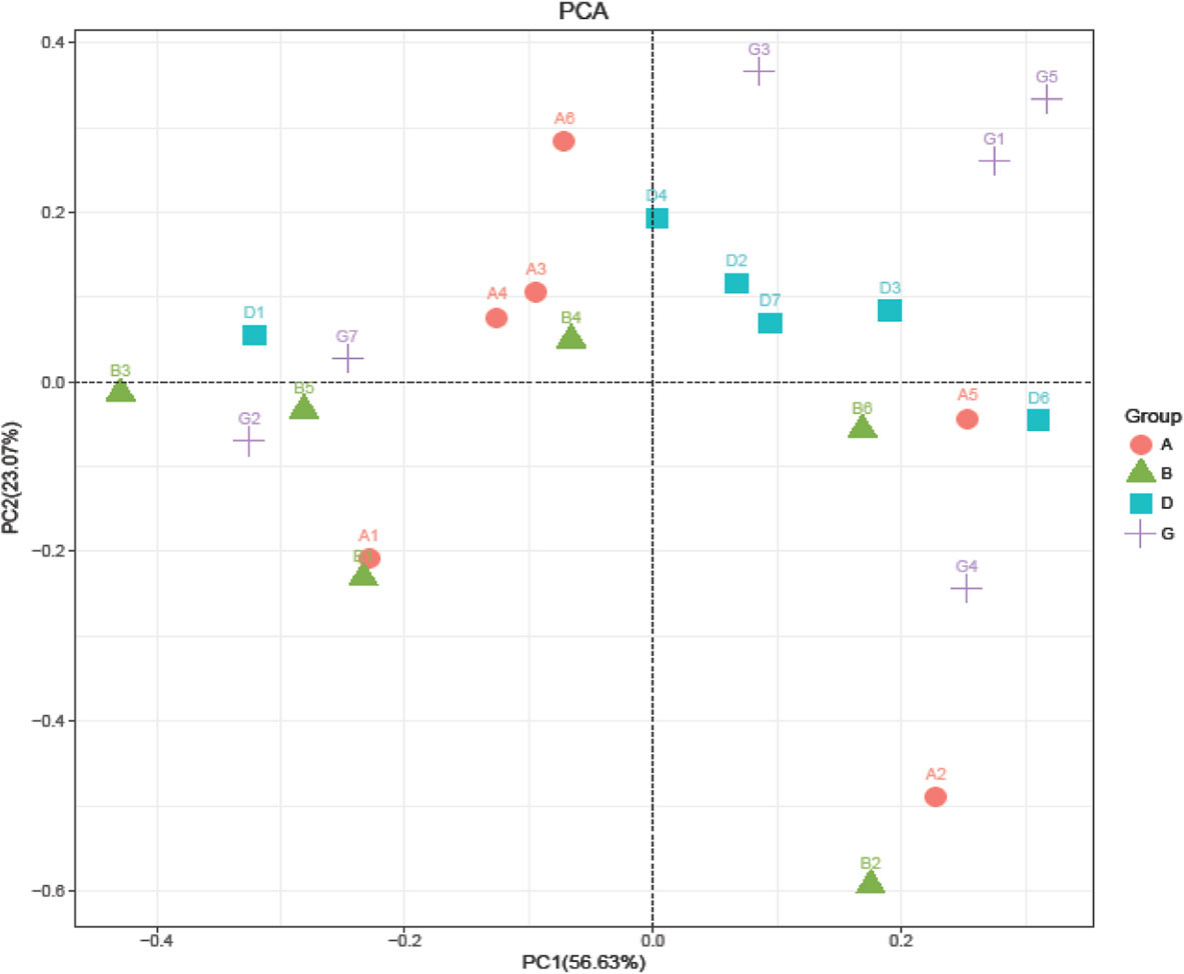
Effect of BLJ on cecal microbiota beta-diversity of the broiler chickens subjected to SNE challenge. **a** = a basal diet + unchallenged; **b** = a basal diet with 500 mg/kg of BLJ + unchallenged; **d** = a basal diet + challenged; and **g** = a basal diet with 500 mg/kg of BLJ + challenged

**Fig. 3:**
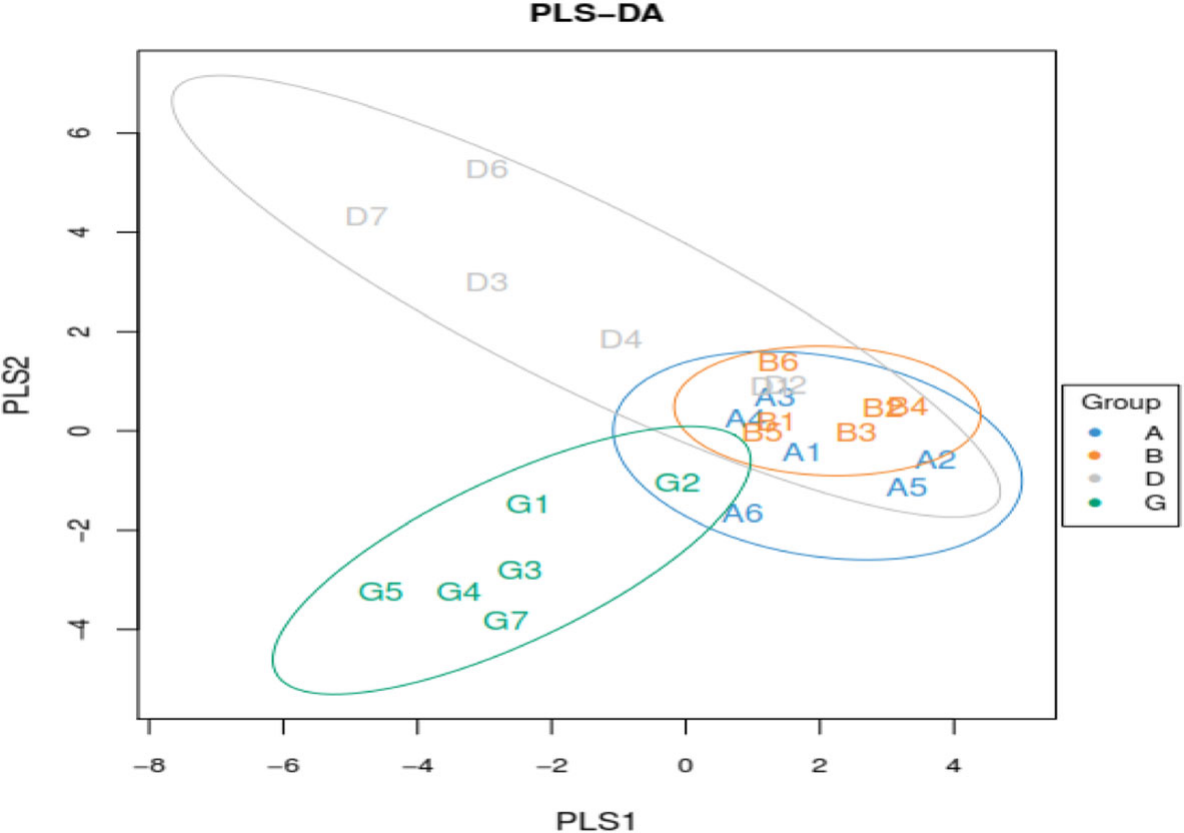
Partial least squares discriminant analysis (PLS-DA) scores derived from cecal microbiota of broiler chickens infected with NE (indicating the degree of reliability of PCA analysis). (Difference of cecal microbiota relative abundance at a general level). **a** = a basal diet + unchallenged; **b** = a basal diet with 500 mg/kg of BLJ + unchallenged; **d** = a basal diet + challenged; and **g** = a basal diet with 500 mg/kg of BLJ + challenged

To study the effect of the BLJ on the gut microbiotas of broiler chickens infected with NE, the cecal contents of the microbiome were analyzed via deep sequencing. In this study, 769274 effective and high-quality sequences were obtained from all samples (*n* = 24) after processing and filtering. The average coverage of each sample was 45685 (range, 34585–91303) reads. These OTUs were generated and characterized for different taxonomic levels, including phylum and genus, as per the Greengenes database using QIIME. A Venn diagram of the OTU numbers indicated 1776 common core OTUs for all groups and 209, 309, 382 and 235 unique OTUs for the four groups (Fig. 1). Alpha diversity measured using the ACE, Chao1, Simpson and Shannon indices showed that community richness and diversity for the cecal feces did not differ (*P* > 0.05; Table 9) among the groups, indicating that NE infection, BLJ treatment or their combination did not alter the alpha diversity of the cecal microbial diversity. Principal component analysis revealed that individuals from each group appeared to be interspersed (Fig. 2), indicating significant variability in the cecal microbiota composition and structure between these groups. Whereas PLS-DA scores for the cecal microbiota showed that the microbial communities were distinctly separated between the untreated NE-infected birds and the NE-infected BLJ-treated birds (Fig. 3).

**Table 9:** Effect of BLJ on cecal microbiota α-diversity of the broiler chickens subjected to NE challenge

Items	NE^1^	Simpson	Chao 1	ACE	Shannon
BLJ, mg/kg					
0	–	0.91	1747.37	1807.71	6.51
0	+	0.93	1625.39	1656.49	6.92
500	–	0.91	1725.20	1830.70	6.70
500	+	0.95	1728.87	1856.10	7.19
SEM^2^		0.01	49.89	55.50	0.14
Main effect					
0		0.92	1686.38	1732.10	6.72
500		0.93	1727.03	1847.90	6.95
Non-challenged		0.91	1736.08	1819.20	6.61
Challenged		0.94	1677.13	1760.80	7.05
*P*-value^3^					
BLJ		0.723	0.702	0.320	0.402
Challenge		0.179	0.579	0.613	0.113
Challenge×BLJ		0.679	0.556	0.423	0.878

^1^*NE* Co-challenged with *Eimeria* spp. and *Clostridium perfringens*; −, without NE challenge; +, with NE challenge

^2^*SEM* standard error of the mean

^3^*P*-values represent the main effect of the diet, the main effect of the NE challenge, and the interaction between the dietary treatments and NE challenge

**Fig. 4:**
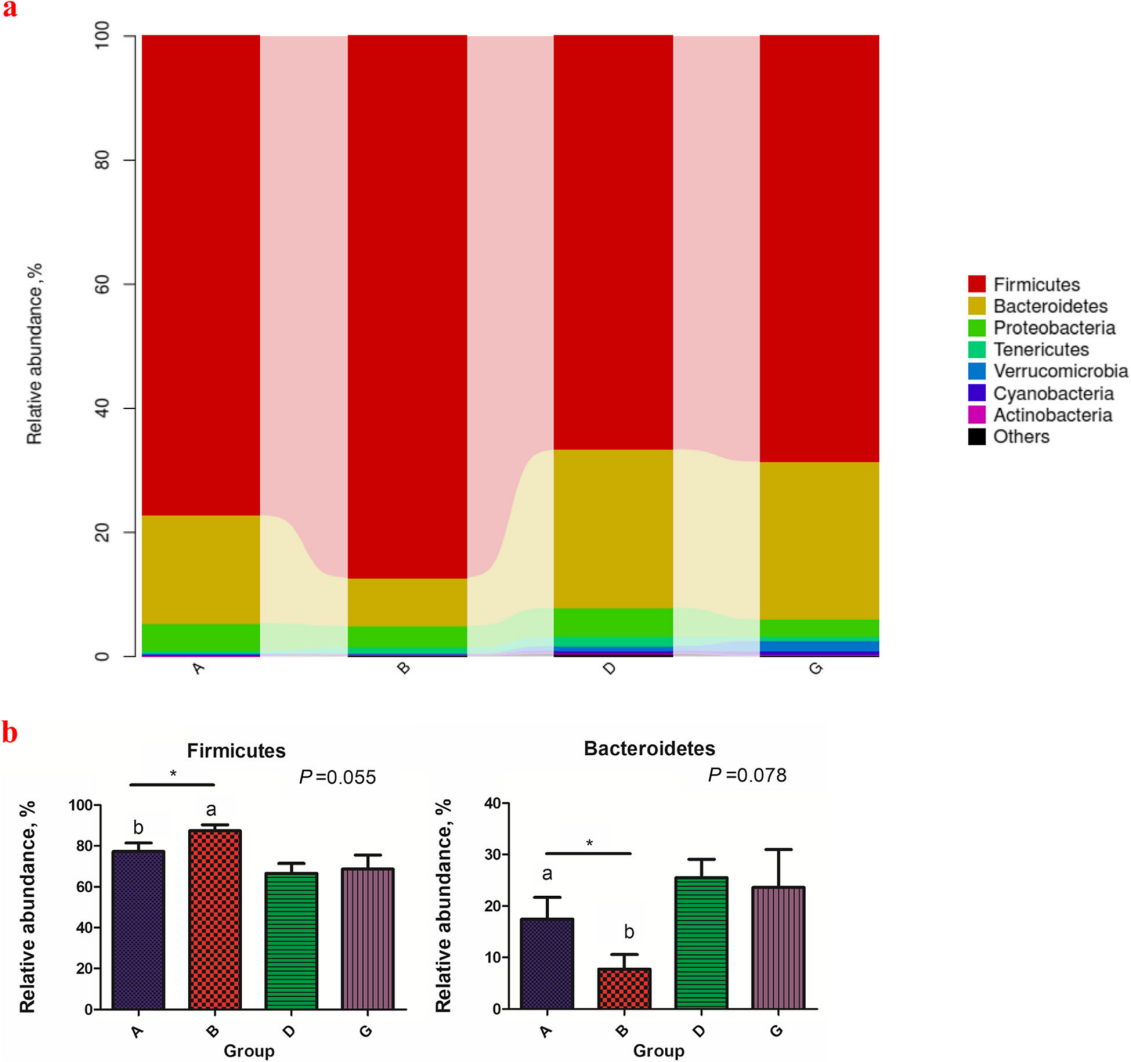
Effects of BLJ on composition of cecal microbiota at the phylum levels. **a)** Composition of caecal microbiota of the broiler chickens at phylum level. **b)** Comparison of the relative abundances of the two main bacterial phyla. A = a basal diet + unchallenged; B = a basal diet with 500 mg/kg of BLJ + unchallenged; D = a basal diet + challenged; and G = a basal diet with 500 mg/kg of BLJ + challenged

**Fig. 5:**
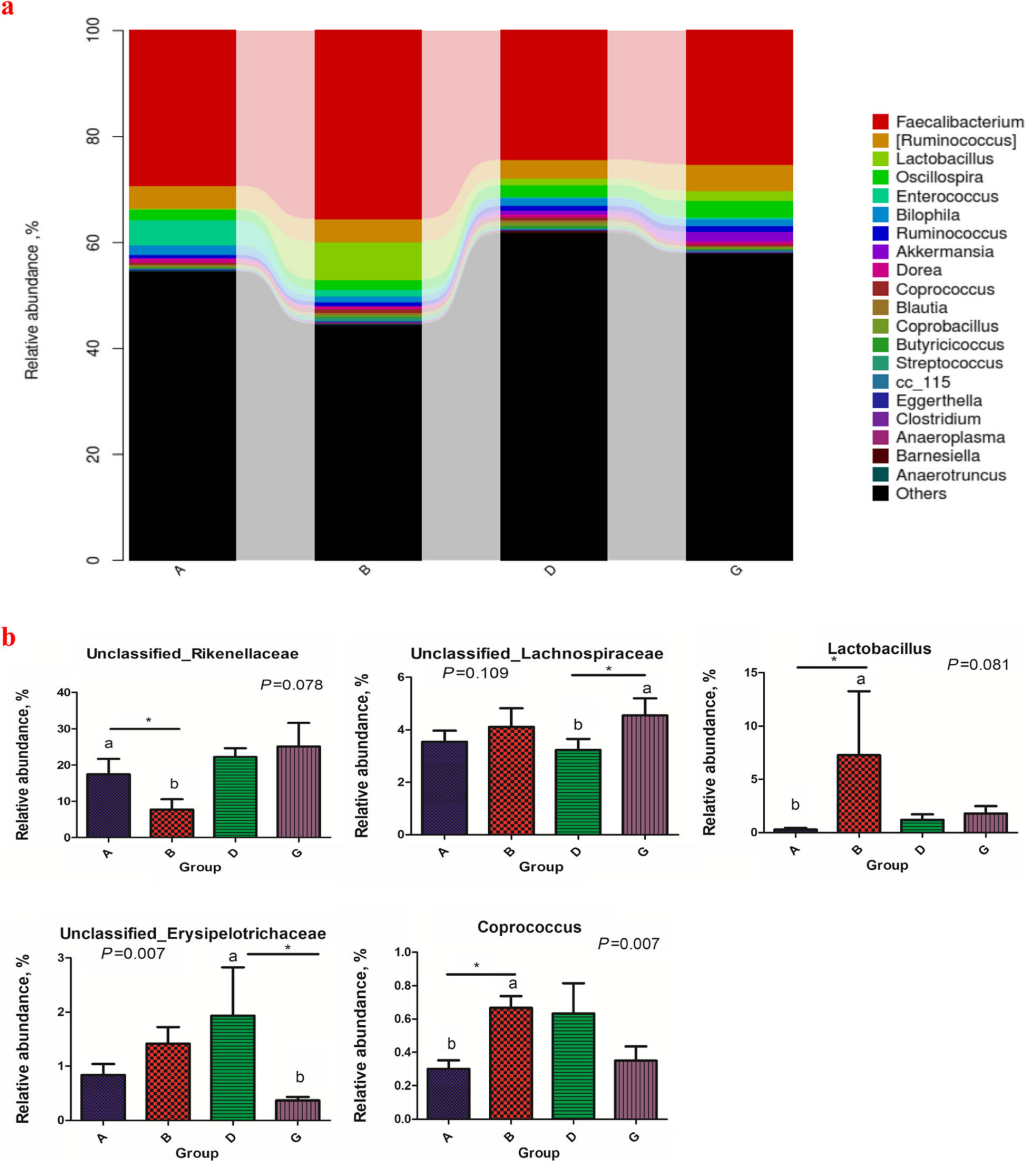
Effect of BLJ on cecal microbiota relative abundance (at a general level) of broiler chickens challenged with NE. **a)** Overall fecal microbiota composition of the samples at the genus level. **b)** Comparison of the relative abundances of the five bacterial genera. A = a basal diet + unchallenged; B = a basal diet with 500 mg/kg of BLJ + unchallenged; D = a basal diet + challenged; and G = a basal diet with 500 mg/kg of BLJ + challenged

To investigate the impacts of BLJ supplementation on the cecal microbial compositions of the NE-challenged birds, we compared the variation in bacterial taxa between the A and B and the D and G groups. Phylum level analysis demonstrated that NE infection, BLJ treatment and their combination (*P* < 0.05) influenced the percentages of Firmicutes and Bacteroidetes but not the relative abundances of Proteobacteria, Actinobacteria or other bacterial phyla on d 28 (Fig. 4). However, under the unchallenged conditions, BLJ addition exhibited an increased Firmicutes abundance (*P* = 0.055) and a reduction in Bacteroidetes (*P* = 0.078). For the NE-challenged birds, those fed BLJ did not differ significantly in the relative abundances of phyla. At the genus level, compared with the uninfected and untreated birds, the uninfected birds fed BLJ displayed increased relative distributions of *Lactobacillus* (*P* = 0.081) and *Coprococcus* (*P* = 0.007) but decreased Rikenellaceae (*P* = 0.078) levels (Fig. 5). For the NE-challenged birds, infected birds fed BLJ showed increased relative abundances of Unclassified_Lachnospiraceae (*P* = 0.109) and significantly decreased relative abundances of Erysipelotrichaceae (*P* = 0.031).

## Discussion

In the present study, NE challenge decreased the BWG and AFI and increased the feed/gain ratio in chicks fed a basal diet during the infection period, which is consistent with the results reported by Song et al. [28] and Wu et al. [37]. Birds fed a commercial blend of thyme, carvacrol and organic acids (BLJ) showed improved FCR and decreased AFI but no statistical differences in BWG regardless of NE infection. Our results indicated that feeding with BLJ could positively improve FCR in broilers. Consistent with our findings, several recent studies documented that EOA improved the growth performance and/or feed utilization in broilers [47, 48] and turkeys [49]. Conversely, other studies suggested that the blend of EOs and sodium butyrate yielded no significant difference in growth performance [50]. These inconsistencies in the efficacy of the EOAs on growth performance may be related to differences in the composition and inclusion levels used, diet type, environmental conditions, animal age, breed and hygienic conditions between studies [3]. Poor hygienic conditions may help the EOs to favorably affect the growth performance and feed efficiency of broilers [51].

Intestinal morphology, including villus height, crypt depth and the VH/CD ratio, is an important indicator of intestinal health, recovery and functionality and plays a significant role in nutrient digestion and absorption [52]. In the current study, we further investigated the effect of adding BLJ on intestinal health of the NE-challenged broilers to explain the mechanisms by which BLJ improves FCR. NE infection alone led to higher gut lesions, atrophied villus height, longer crypt depths and a reduced VH/CD ratio; however, these changes were reversed, attenuated or alleviated by including BLJ in the diet, thus indicating that BLJ addition alleviated the mucosal atrophy and epithelial cell necrosis due to NE challenge. Consistent with our findings, broilers infected with *C. perfringens* and treated with combined sodium butyrate and EOs (ginger oil and carvacrol) protected with vegetable fat also yielded better BWG, increased villus lengths and VH/CD ratios and decreased gross pathological and histopathological lesion scores compared with those of the controls [53]. Two previous studies demonstrated that feeding blends of sorbic acid, fumaric acid and thymol to broiler chickens reared under conventional conditions changed the intestinal morphology, resulting in longer villi and a greater VH/CD ratio [35, 47]. In addition, several studies have shown improved gut morphology and decreased gross intestinal lesion scores in NE-infected broiler chickens fed either EO-supplemented [18] or OA-treated diets [28]. Intestinal bacterial translocation to internal organs and serum FITC-D levels post-FITC-D gavage are also two important parameters commonly used to indirectly evaluate quality and extent of intestinal injury [54]. Our study further found that infected birds fed BLJ showed lower *C. perfringens* loads in the liver and cecal contents and decreased serum FITC-D concentrations after oral administration of FITC-D in contrast to the NE-infected birds. These results indicated that including dietary BLJ inhibited *C. perfringens* growth, colonization and translocation and improved intestinal barrier integrity against NE-induced gut barrier injury in broiler chickens under NE infection. Likewise, previous studies also reported that the EOA combination can decrease *C. perfringens* colonization and proliferation in chicken guts [54]. Improved gut morphology, reduced gut lesion scores and decreased intestinal epithelial permeability in NE-infected birds that received BLJ were possibly attributed to the antibacterial traits of the EOAs [34], EOs [11] or OAs [25]. The beneficial effect on FCR in the BLJ-fed birds with or without NE infection may have been attributed to gut health improved by BLJ administration. Therefore, our results suggested that BLJ appeared to be effective in lessening the NE lesion severity.

The intestinal barrier is regulated by tight junction proteins (TJPs) that consist of several unique proteins, including the junction adhesion molecule, the transmembrane protein, occludin, members of the claudin family and linker proteins such as the zonula occludin protein family (ZO). This mechanical barrier plays an important role in the absorption of nutrients, electrolytes and water, as well as maintaining intestinal-barrier integrity and function and protecting the gut from enteric pathogen invasion. Intestinal TJP barrier disruption leads to endogenous infection and sustained inflammation and tissue damage, and it reduces nutrient absorption [55]. In this study, NE infection remarkably downregulated occludin and *ZO-1* mRNA levels, while the NE-infected birds fed BLJ showed upregulated claudin-1 mRNA expression levels in the jejunum compared with those of untreated NE-infected birds. These results were consistent with previous findings in broilers fed EOAs [15]. Additionally, increased TJP gene expression and improved intestinal barrier function were observed in EO (thymol and carvacrol)-treated broilers challenged with *C. perfringens* [56]. Furthermore, birds that received BLJ showed upregulated mucin-2 mRNA levels compared with those of the unsupplemented groups, regardless of NE infection. Claudin-1 is a member of the multiple-spanning, transmembrane, claudin protein family, which plays important roles in barrier formation and paracellular selectivity in various tissues [57]. Mucin-2, secreted by goblet cells, covers the intestinal epithelial surface and plays a major role in protecting the intestinal epithelium from infection and maintaining intestinal mucosal barrier integrity, immune hemostasis and gut health [58]. Here, increased claudin-1 gene expression was observed in the jejunums of NE birds administered BLJ, indicating that BLJ addition helps protect the gut barrier from direct contact with pathogenic bacteria in NE-infected broilers. Enhanced TJPs following BLJ supplementation resulted in reduced gut permeability and pathogen invasion. Thus, our results also suggested that BLJ, a protective EOA blend, may help maintain gut health. Interestingly, we also found that dietary BLJ supplementation significantly downregulated *ZO-1* expression levels in the jejunum compared with those of the unsupplemented controls regardless of NE challenge. In contrast with previous results, broiler chickens fed carvacrol EOs or coated butyrate exhibited upregulated *ZO-1* and *ZO-2* mRNA levels in the intestinal mucosa [15]. The ZO proteins, ZO-1, ZO-2 and ZO-3, are important molecules that interact directly with occludin, claudins and actin thereby providing a scaffold that facilitates regulation of the expression and distribution of the TJP complex [59]. The changes suggested that BLJ supplementation differentially regulated TJP complex expression and distribution in the gut but did not damage or alter its intact structure. The reason that BLJ downregulates *ZO-1* requires further investigation.

TLR-mediated signaling pathways are involved in regulating intestinal epithelial barrier integrity [60]. In our study, NE infection significantly increased *IFN-γ* and *IGF-2* mRNA levels and downregulated *TLR-4,* adaptor protein tumor necrosis factor receptor (TNFR)-associated factor 6 (*TRAF-6*)*, NF-κB, TNFSF15, TLR*-activating negative regulators *TOLLIP*, *PI3K* and *SOCS-6* and *EGFR* mRNA expression in the jejunum compared with the unchallenged control. Suppression of negative regulators of the TLR signaling pathway suggested that the TLR signaling pathway was activated, resulting in inflammatory cytokine production. Upregulated *IFN-γ* gene mRNA levels were observed in the untreated NE-infected birds, which were consistent with previous studies [61]. These results showed that NE infection differentially modulated intestinal immune-related gene and growth factor gene expression, thus activating intestinal immuno-inflammatory responses. NE infection markedly increased intestinal inflammation, possibly by increasing proinflammatory cytokine *IFN-γ* gene expression, whilst downregulating *TLR*-activating negative regulators in broilers. However, dietary BLJ supplementation remarkably downregulated *TLR-4* and *TRAF-6* gene expression levels, decreased *IL-1β* gene expression levels and increased *A20* and *SOCS-6* mRNA levels in the jejunal mucosa of broilers regardless of NE infection. This result suggested that BLJ showed anti-inflammatory functions in the chicken intestines by upregulating negative-factor *SOCS-6* and *A20* gene expression and inhibiting *TLR4*-mediated signal pathway activation. Additionally, NE-infected birds fed diets containing BLJ displayed decreased relative gene expression of *TRAF-6*, *TNFSF15* and *TOLLIP* and reduced *TLR2* gene expression levels but displayed increased *IGF-2* mRNA levels in the jejunum compared with those in the untreated NE-infected birds. Proinflammatory cytokines, such as TNF-α, IFN-γ and IL-1β, are reported to increase intestinal permeability and tissue damage by dysregulating TJPs [62], while various regulatory peptides including anti-inflammatory cytokines (TGF-β, IL-4 and IL-10), growth factors (EGF, GLP-2 and IGF-2) and negative regulators (A20, SOCS, TOLLIP and PI3K) of the TLR signaling pathway protect intestinal barrier function by regulating TJP expression and facilitating repair of damaged gut tissue [63]. Here, NE infection compromised the intestinal epithelial barrier integrity, possibly associated with intestinal immuno-inflammatory responses, while suppressing *TLR-2*, *TRAF-6* and proinflammatory cytokine *TNFSF15* mRNA and upregulating growth factor *IGF-2* mRNA via BLJ in the intestines of the BLJ-fed chickens following NE infection. This result indicated that BLJ pretreatment could reduce the progress and development of intestinal inflammation, alleviate NE-induced intestinal inflammation, improve gut health and protect the intestinal barrier structure as evidenced by the attenuated gut lesions, reduced bacterial translocation to the liver and increased VH/CD ratios in the jejunum. The anti-inflammatory effect of BLJ has generally been attributed to the antimicrobial and immune-regulating actions of the EOs [17] or OAs [64] in the BLJ. The reduced intestinal inflammation may eventually lead to improved gut health and FCR in BLJ-treated chickens, possibly by modifying the TLR-mediated signaling pathway.

The gut microbiota constitutes a highly complex ecosystem that interacts with the host and profoundly affects the physiological, immunological, nutritional and metabolic status of the host [65, 66]. To further investigate the mechanism underlying BLJ mitigating NE-induced gut injury, the cecal microbiota structure was analyzed via Illumina MiSeq sequencing. This study revealed no differences in α-diversity of the cecal microbiota between the four experimental groups, which was consistent with previous results [67]. In addition, principal component analysis showed that BLJ supplementation, NE challenge or both altered the β-diversity of the cecal microbiota, indicating that these treatments significantly affected the intestinal bacterial community profiles. However, NE challenge reduced the relative abundance of the phylum Firmicutes (67.65% vs. 82.27%) and increased the relative levels of Bacteroidetes compared with those of the unchallenged group, which helps explain the impaired BWG in the NE-infected birds, consistent with previous research [67]. We also found that the relative abundance of Firmicutes increased, while the relative abundance of Bacteroidetes decreased after BLJ treatment in unchallenged birds. Increases in fecal Firmicutes have been associated with increased nutrient absorption [68] and body weight gain [69], whereas increases in fecal Bacteroidetes have been associated with decreased nutrient absorption [67, 70]. Therefore, a higher abundance of Firmicutes might enhance energy absorption in birds fed BLJ, resulting in the improved FCR observed in our study.

Infected birds administered BLJ showed an increased percentage of Unclassified_Lachnospiraceae and a significantly decreased relative abundance of Erysipelotrichaceae. Additionally, compared with the untreated uninfected birds, the uninfected birds fed BLJ displayed increased relative abundances of *Lactobacillus* and *Coprococcus* and fewer Rikenellaceae. Similarly, previous studies have shown that EOA blends increased *Lactobacillus* spp*.* counts in the ileal digesta in pigs [71] and chickens [48]. A supplemental EO blend (thymol and carvacrol) increased ileal *Lactobacillus* populations and reduced the effect of NE due to *C. perfringens* in chickens [49]. OAs added to broiler feed can increase Lactobacillus populations and reduce pathogenic bacteria in the gastrointestinal tract [72]. Therefore, our findings suggested that BLJ supplementation altered the cecal microbial communities in broiler chickens, regardless of NE infection. Lactobacillus spp. could inhibit intestinal inflammatory responses and displace pathogenic bacteria including Salmonella and *C. perfringens* from the gut by producing OAs or bacteriocins [73]. Members of the Lachnospiraceae family, including *Coprococcus*, *Roseburia* spp. and *Eubacterium rectale*, were found to have a protective effect in patients with colon cancer by producing n-butyrate [68], they suppressed *C. difficile* in the mouse gut [69] and positively correlated with feed conversion efficiency in broiler chickens [70]. Erysipelotrichaceae abundance has been negatively correlated with body fat weight, the colonic butyrate concentration and intestinal health [74]. Rikenellaceae abundance has been positively correlated with clinical disease severity [75], and a reduced proportion of Rikenellaceae is considered to be a signature of a healthy gut. Recent evidence suggested that butyrate reduces the incidence and severity of NE, thus preventing ascending infections when added to feed [76]. Therefore, higher abundances of *Lactobacillus*, butyrate-producing Unclassified_Lachnospiraceae and *Coprococcus*, and a relatively lower proportion of Erysipelotrichaceae and Rikenellaceae, are present in the intestines of BLJ-treated broiler chickens regardless of NE infection, suggesting that including BLJ into the diets of birds can improve gut health by promoting the growth of potentially beneficial intestinal microorganisms and inhibiting the proliferation of harmful bacteria. Therefore, our study suggested that dietary BLJ benefitted gut health, and the mechanism for this effect may involve altering the gut microbial communities. Further fecal-metabolome analyses are required to explore the impact of BLJ on fecal metabolite profiles. These analyses may enable possible causal links to be established between BLJ, probiotics, metabolites and gut function.

## Conclusions

In summary, the protected EOs and organic acid blends (BLJ) effectively ameliorated NE-induced intestinal injury, possibly by regulating the intestinal microbial communities and differentially modulating the intestinal mucosal immune responses and barrier function. These findings indicate that BLJ may be a potential and promising candidate for preventing NE in broiler chickens.

## Data Availability

All data generated or analyzed during this study are available from the corresponding author by request. The datasets supporting the conclusions of this article are included in the article.

## Abbreviations

A20: Protein A20
AFI: Average feed intake
BWG: Body weight gain
CD: Crypt depth
CFU: Colony-forming unit
EGFR: Epidermal growth factor receptor
FCR: Feed conversion ratio
GLP-2: Glucagon-like peptide-2
IFN-γ: Interferon-γ
IGF-2: Insulin-like growth factor-2
IL: Interleukin
MyD88: Myeloid differential protein-88
NE: Necrotic enteritis
NF-κB: Nuclear factor kappa-light-chain-enhancer of activated B cells
NRC: National Research Council
PI3K: Phosphatidylinositol 3-kinase
SOCS: Suppressor of cytokine signaling
TGF-β3: Transforming growth factor beta 3
TLR: Toll-like receptor
TNFSF15: Tumor necrosis factor superfamily 15
TOLLIP: Toll-interacting protein
TRAF-6: TNF receptor-associated factor 6
VH: Villous height
VH:CD ratio: The ratio of villus height to crypt depth
ZO-1: Zonula occludens-1
